# Transnasal TOE: An alternate approach in the setting of difficult probe placement for seated spinal surgery

**DOI:** 10.4103/0019-5049.60503

**Published:** 2010

**Authors:** Michael G Fitzsimons, Brinda Kamdar, Johnica Eyvazzadeh, B Heidi

**Affiliations:** Harvard Medical School, Division of Cardiac Anesthesia, Department of Anesthesia and Critical Care, Massachusetts General Hospital, Bosten, USA; 1 Medical Anesthesia Consultants Medical Group, Inc., Bosten, USA

**Keywords:** Craniotomy, echocardiography, kyphoscoliosis, transoesophageal echocardiography

## Abstract

Transnasal transoesophageal echocardiography may be an effective alternative approach when difficulty is encountered while placing a probe for patients with severe kyphoscoliosis. We describe a successful approach in a patient presenting for orthopaedic fixation and review the current literature.

## INTRODUCTION

Transnasal placement of a transoesophageal echocardiography (TOE) probe has been previously described in the literature. Indications have included unexpected difficulty with transoral placement,[1] placement in intubated patient[2] and assessment of patent foramen ovale.[3] No review has described a transnasal approach in a situation where difficulty with probe placement is anticipated but necessary for surgery in the sitting position. We describe transnasal placement of a paediatric monoplane TOE probe in a patient with severe ankylosing spondylitis.

## CASE REPORT

A 57-year-old male with a history of ankylosing spondylosis and worsening cervicothoracic kyphosis presented for orthopaedic stabilisation of his cervical spine. Over the two years prior to his presentation he had lost approximately 7 inches of his height despite a prior attempt at fixation. He complained of diffuse pain throughout his entire spine but denied any weakness of the upper or lower extremities. Physical examination revealed a 5 feet, 6 inch 230 Lb male. Vital signs showed a blood pressure of 101/64 mm Hg, heart rate 73 beats per minute and room air oxygen saturation 98%. He had a chin-to-chest deformity and severe kyphosis of the cervical and thoracic spine. Neck extension was limited to approximately 10°. Mouth opening was approximately three-finger-breadth revealing a Mallampatti Class III airway. Examination of the lungs and heart were normal. Laboratory analysis was normal. Chest x-ray demonstrated elevation of the right hemi diaphragm to the level of the hilum but this was stable when compared to prior studies.

**Figure 1:**
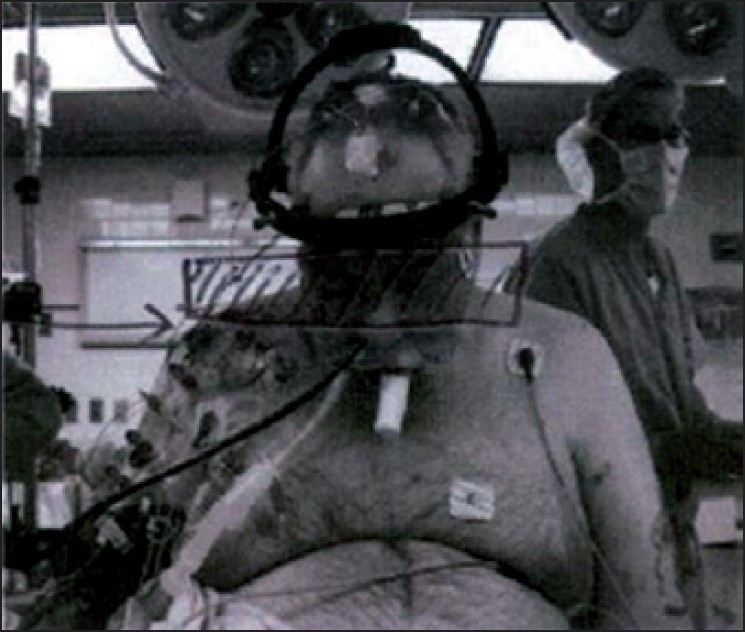
Transnasal TOE probe successfully placed in patient for spine surgery after difficulty found with oral route

After informed consent was obtained from the patient, standard monitors were applied and a 16G intravenous catheter was started. The patient received sedation consisting of 4mg of intravenous midazolam in 2 mg increments. General anaesthesia was induced with a rapid sequence approach. The patient was administered 100% oxygen for five minutes via face mask followed by fentanyl (1 mcg.kg^1^), propofol (2 mg.kg^1^), and succinylcholine (1 mg/kg); the trachea was then intubated fiber optically and lungs were mechanically ventilated (tidal volume 8 ml.kg^1^, respiratory rate 12 breaths/min; inspiratory to expiratory time 1:2). Total intravenous general anaesthesia (TIVA) using remifentanil (0.15-1 mcg/kg/min), propofol (50-100 *μ*g/kg/mm) and ketamine (5-10 *μ*g/kg/min) was selected to facilitate use of intraoperative motor and sensory evoked potentials. Hydromorphone was administered as needed for analgesia in increments of 1-2 mg boluses for a total of 15 mg. Routine monitoring included electrocardiogram (ECG), heart rate (HR), temperature, end tidal carbon dioxide tension (EtCO_2_), hourly urinary output, and haemoglobin oxygen saturation (SpO_2_). Arterial and central venous catheters were inserted for monitoring of arterial and central venous pressures and for the potential use of inotropes or catecholamine. Difficulty was encountered placing an adult TOE probe via the oral route. A paediatric echocardiography probe (Phillips Medical Systems, Bethel, WA) was easily inserted into the right nostril and advanced without trauma into the oesophagus [Figure 1]. Routine images were easily obtained. Ventricular function was normal, valves normal, and there was no evidence of a patent foramen ovale. The case proceeded uneventfully and the patient made a full recovery.

## DISCUSSION

The presence of a patent foramen ovale in a patient presenting for a craniotomy in the sitting position increases the risk of paradoxical vascular air embolism (PAE). Vascular air embolism may occur in nearly 10% of neurosurgical cases performed in the sitting position.[4] Haemodynamic complications may include tachyarrhythmias, right heart strain, myocardial ischaemia, hypotension, and a reduction in cardiac output.[5] Neurologic compromise including mental status changes and ischaemia may result after PAE.

TOE may be the most sensitive indicator of the presence of air in the cardiac chambers; a volume as little as 0.02 mL/kg may be detected.[6]

The incidence of failure to pass a TOE probe is extremely low in anaesthetized patients (0.18%).[7] The causes of failure are unclear but may include oesophageal abnormalities or operator inexperience. The nasal route may be a viable primary alternative in certain patients including those with severe cervicothoracic kyphosis.

The advantages of the nasal route may include better tolerance in the awake patient and easier intubation when a probe is needed prior to induction of general anaesthesia. Greim performed 60 TOE placements in awake patients scheduled for posterior fossa neuro-surgery.[3] Placement was successful in 56 of 60 patients. Detection of a PFO in the awake patient with a Valsalva manoeuvre was possible in seven of 56 patients. Seven additional patients were identified as having a PFO after intubation with a manual “Valsalva” manoeuvre. Griem also studied transnasal TEE in 42 intubated patients. Placement was successful in 90% of the cases.[2] Zimmermann reported a study of placement of a transnasal TOE probe prior to induction of anaesthesia in 45 patients.[8] Probe placement was successful in 43 of 45 patients. Induction of general anaesthesia occurred. The ability to provide mask ventilation was “good” in 40/42 patients and intubating conditions “good” in 37/42 patients.

A potential disadvantage of the transnasal route is potentially inferior imaging. Griem did reported that although the IV short-axis and four-chamber views could be easily obtained, the smaller probe required for the transnasal route lacked multiplane capability and other images were inferior to multiplane imaging.[2] Spencer *et al*. studied placement of miniaturised, monoplane probe. Probe placement was successful via the nasal route in 63/75 patients. Failure was primarily related to the inability to pass the probe through the nasal passage. General image quality was inferior to that obtained with conventional multiplane transducers but assessment was still considered good.

The transnasal route or placement of a TOE probe is safe and effective. The approach may be beneficial in patients with cervicothoracic kyphosis in which the oral route may be difficult. Transnasal TOE will allow satisfactory assessment of the heart in the four-chamber and LV short-axis view but monoplane capability common in the smaller probe may limit more accurate assessment.

## References

[1] Aronson LA (2003). Transnasal placement of biplane transesophageal echocardiography probe intraoperatively in an adolescent with congenital heart disease. Anesth Analg.

[2] Greint CA, Brederlau J, Kraus K, Apfel C, Thiel H, Roewer N (1999). Transnasal transesophageal echocardiography: A modified application mode for cardiac examination in ventilated patients. Anesth Analg.

[3] Greim CA, Trautner H, Kramer K, Zimmerman P, Apfel C, Roewer N (2001). The detection of interatrial flow patency in awake and anesthetized patients: A comparative study using transnasal transesophageal echocardiography. Anesth Analg.

[4] Leslie K, Kaye AE (2006). Venous air embolism and the sitting position: A case series. J Clin Neurosci.

[5] Mirski MA, Lele AV, Fitzsimmons L, Toung (2007). Diagnosis and treatment of vascular air embolism. Anesthesiol-ogy.

[6] Furuya H, Suzuki T, Okumura F, Kishi Y, Uefuji T (1983). Detection of air embolism by transesophageal echocardingraphy. Anesthesiology.

[7] Kallmeyer IJ, Collard CD, Fox JA, Body SC, Sheman SK (2001). The safety of intraoperative transesophageal echocardiography: A case series of 7200 cardiac surgical patients. Anesth Analg.

[8] Zimmermann P, Griem C, Trautner H, Sagmeister U, Kraemer K, Roewer N (2003). Echocardiographic monitoring during induction of general anesthesia with a miniaturized esophageal probe. Anesth Analg.

[9] Spencer KT, Krauss D, Thum J, Mor-Avi V, Poppas A (1997). Transnasal transesophageal echocardiography. J Am Soc Echocardiogr.

